# Validation of Two Activity Monitors in Slow and Fast Walking Hospitalized Patients

**DOI:** 10.1155/2022/9230081

**Published:** 2022-05-16

**Authors:** Britt Stævnsbo Pedersen, Morten Tange Kristensen, Christian Ohrhammer Josefsen, Kasper Lundberg Lykkegaard, Line Rokkedal Jønsson, Mette Merete Pedersen

**Affiliations:** ^1^Department of Clinical Research, Copenhagen University Hospital, Amager and Hvidovre, Denmark; ^2^Copenhagen Neuromuscular Center, Rigshospitalet, Copenhagen, Denmark; ^3^Physical Medicine and Rehabilitation Research-Copenhagen (PMR-C), Denmark; ^4^Department of Orthopedic Surgery, Copenhagen University-Hospital Amager and Hvidovre, Denmark; ^5^Department of Clinical Medicine, University of Copenhagen, Denmark; ^6^Department of Physical and Occupational Therapy, Copenhagen University Hospital-Bispebjerg and Frederiksberg, Denmark; ^7^Department of Physical and Occupational Therapy, Copenhagen University Hospital-Amager and Hvidovre, Denmark; ^8^SENS Innovation ApS, Copenhagen, Denmark

## Abstract

To evaluate interventions to promote physical activity, valid outcome measures are important. This study evaluated the validity and reliability of the ActivPAL3™ and the SENS motion® activity monitors with regard to the number of steps taken, walking, and sedentary behavior in hospitalized patients (*n* = 36) (older medical patients (+65 years) (*n* = 12), older patients (+65) with acute hip fracture (*n* = 12), and patients (+18) who underwent acute high-risk abdominal surgery (*n* = 12)). Both monitors showed good (≥60%) percentage agreement with direct observation for standing and no. of steps (all gait speeds) and high agreement (≥80%) for lying. For walking, ActivPAL3™ showed moderate percentage agreement, whereas SENS motion® reached high percentage agreement. The relative reliability was moderate for sedentary behavior for both monitors. The ActivPAL3™ showed poor (walking) to moderate (steps) reliability for walking and steps, whereas SENS motion® showed moderate reliability for both activities. For slow walkers, the relative reliability was moderate for SENS motion® and poor for ActivPAL3™. This trial is registered with the ClinicalTrials.gov identifier NCT04120740.

## 1. Introduction

Hospitalization is often associated with inactivity and bedrest [1–5], which can lead to functional decline, loss of independence, complications (e.g., thrombosis, pneumonia, or intestinal obstruction), mortality, and increased risk of rehospitalization [6–9]. Newly developed recommendations for physical activity during hospitalization state that older adults should be as active as possible during hospitalization and that long periods of sedentary behavior during waking hours should be minimized [10]. Also, it has been shown that daily ambulation during hospitalization can reduce the risk of loss of mobility [11]. Therefore, interventions that are aimed at reducing sedentary time and increasing levels of activity during hospitalization are needed. To evaluate such interventions, valid outcome measures are important. Accelerometers can be used to measure and classify physical activities [12], as well as motivate to physical activity [13]. One type of accelerometer is the ActivPAL3™ [14], which can measure physical activities (lying, standing, and walking). The ActivPAL3™ accelerometer has proven valid and reliable in measuring postures (standing and lying/sitting) in adults of 35-55 years [15] and 18-65 years [16] and older adults of 72-87 years with stroke and hip fracture as well as geriatric inpatients [17]. Also, the ActivPAL3™ is valid and reliable in measuring walking (number of steps) at gait speeds ≥ 0.90 m/s in healthy young adults (25-35 years) [18] and at gait speeds ≥ 0.67 m/s in healthy adults (18-39 years) [19] and in community-dwelling older adults (65-87 years) without mobility impairments [20]. However, the ability of the ActivPAL3™ to accurately assess walking at slower gait speeds in mobility-impaired older adults is uncertain [17, 21]. The SENS motion® system is a newer activity monitor system that is valid in measuring sedentary behavior in patients with knee osteoarthritis [22]. In 2016, some of the authors of the present study performed a small pilot study (*N* = 10) to investigate the accuracy of SENS motion® in older medical patients compared to direct observations, and an 80% accuracy of the SENS motion® was found. However, the SENS motion® algorithm for measuring walking and stepping has been further developed, and the present version has not yet been evaluated in older medical patients and other patient groups. Therefore, the aim of this study was to examine the criterion validity and the reliability of the ActivPAL3™ and the SENS motion® systems in hospitalized patients, by examining the activity monitors' ability to measure the number of steps taken, as well as walking and sedentary time in comparison with direct observations.

## 2. Methods

### 2.1. Study Design

The study is a cross-sectional, observational study conducted to examine the criterion validity and reliability of two different activity monitors, ActivPAL3™ (ActivPAL) and SENS motion® (SENS) used as both single- and dual-sensor systems. The study was performed from October 2019 to November 2019 at XXX Hospital.

### 2.2. Participants

We included three convenience samples of patients admitted to the hospital from their own home: older medical patients (+65 years) (MED) (*n* = 12), older patients (+65 years) with acute hip fractures (*n* = 12), and adult patients (+18 years) who underwent acute high-risk abdominal surgery (AHA surgery) (*n* = 12). Each morning lists of eligible patients referred to physiotherapy were provided by health care professionals at the relevant departments. Patients were excluded on the following criteria: inability to walk independently with or without a walking aid, inability to transfer from lying to sitting position, inability to transfer from sitting to standing, inability to cooperate, and inability to speak or understand Danish. Two of the authors (BSP and COJ), who are both physiotherapists, were responsible for inclusion, assessments, and data collection. All patients gave written informed consent before inclusion. According to the Regional Ethics Committee, the project did not require approval by the Regional Ethics Committee (journal no. H-19031296). Handling of data was approved by the Danish Data Protection Agency (journal no. P-2019-555).

### 2.3. Procedures

The included patients were asked to perform a set of standardized activities while simultaneously wearing both the ActivPAL and the SENS sensors. Each activity was predefined to last a specific number of minutes (Table 1), and the order of the activities was randomized. The time spent in each activity was also measured by direct observation using a stopwatch, which was considered the golden standard compared to activity measured by the included activity monitors. For each patient, all data were collected at the same assessment session, lasting between 30 and 60 minutes.

### 2.4. ActivPAL3™ (ActivPAL)

ActivPAL micro (PAL Technologies Ltd, Glasgow, UK) is a single sensor activity monitor aimed at classifying activities such as lying/sitting, standing, walking, transitions, and number of steps taken. ActivPAL was worn halfway between the spina iliaca anterior superior and the patella on the front side of the right thigh. For attachment, the monitor was covered in Tegaderm™ transparent waterproof film (3 M, Maplewood, MN, USA) and was attached to the patient with a PALstickie™ (dual-layer hydrogel adhesive pad). ActivPAL is a triaxial accelerometer, which samples at 20 Hz and registers its orientation and acceleration. Based on the sensor's orientation and acceleration, a predefined algorithm categorizes the recordings in predefined activities, e.g., sitting and lying down, standing, walking, and number of steps. Walking cadence below 0.3 Hz will be categorized as standing. Raw data are stored in the device and can be downloaded using ActivPAL software. We downloaded and used 15 s epoch files for the analyses. For this study, two ActivPAL sensors were used to be able to equally compare data from ActivPAL with SENS data. The second ActivPAL sensor was placed just below the sternal end of the left clavicle.

### 2.5. SENS motion® (SENS)

SENS (SENS Innovation ApS) is a waterproof activity sensor (45 × 23 × 5 mm, 6 g) to be worn laterally on the right thigh approximately 10 cm above the lateral epicondyle. The sensor is placed in a small patch specially designed for the SENS sensor and is attached on the skin. The sensor is a triaxial accelerometer, which samples at 12 Hz and registers the orientation and acceleration of the thigh. The sensor connects wirelessly to a smartphone application, and the recorded data are transmitted to the application every 10 minutes, when the smartphone is within network reach or stored on the sensor for later transmission. The raw triaxial accelerometer data are transferred automatically to a secure web server via the smartphone. Based on the sensor's orientation and acceleration, a predefined algorithm categorizes the recordings in predefined activities, e.g., sitting and lying down, standing, walking, and number of steps. For SENS, upright motion below 0.2 Hz or noncontinuous walking during the sample epochs (5 seconds) is categorized as sporadic walking. SENS is used primarily as a single sensor system, but a dual-sensor algorithm has been developed to enable distinction between different sedentary positions, including sitting with inclined and upright backrest. Therefore, for this study, two sensors were used to evaluate the accuracy of both a single sensor worn on the thigh and a dual-sensor system with a thigh-worn and a chest-worn sensor. The second SENS sensor was placed just below the sternal end of the right clavicle.

### 2.6. The Assessment Protocol

The standardized activities consisted of the following seven activities: lying supine legs straight and backrest flat (1 min), lying supine knees and hips flexed (1 min), sitting in bed legs straight and backrest 60^○^ (1 min), sitting upright on the bed (1 min), sitting on a chair (1 min), standing (1 min), and walking (3 min) (Table 1]. During the 3-minute walking, the number of steps taken was observed. The activities were chosen to investigate the two monitors' ability to classify both active and sedentary behavior. For each patient, the order of the activities was based on computer randomization and assessment of the activities followed a standardized protocol (Appendix 1).

Before assessment, the two sets of sensors were attached to the patient's leg and chest by one researcher (COJ) while a second researcher (BSP) registered the starting time and attachment time of the sensors. Hereafter, the patients were instructed to perform the seven standardized activities guided by researcher one (COJ) to ensure that all activities were performed according to the protocol. Researcher two (BSP) observed the patients during the activities and recorded the exact starting and finishing time of each activity by a stopwatch and counted the number of steps taken with a hand tally (Mitsutomo®). All recordings were noted on a test sheet with a unique ID identification number.

### 2.7. Patient Characteristics

Information on age and sex was collected from the patients' medical records. To assess the patients' gait speed and chair-stand ability, a 4-meter gait speed test [23, 24] and a 30-second chair stand test [25] were performed.

#### 2.7.1. 4-Meter Habitual Gait Speed

To assess habitual gait speed (m/s), the 4-meter gait speed test [23, 24, 26, 27] was performed. The test was performed after the standardized activities and was carried out at the hospital ward. A 4-meter course was marked on the floor, and the patients were asked to walk at their habitual gait speed from a standing start and past the end of the 4-meter course. The use of a walking aid was allowed if necessary [23].

#### 2.7.2. 30-Second Chair-Stand Test

The 30-second chair-stand test was performed after the 4-meter gait speed test. Standard chairs with armrests (45 cm high) were used. With the arms crossed and without using the armrests, the patients were asked to stand up and sit down as many times as possible within 30 seconds [25]. A modified chair-stand test allowing the use of the armrests was used for patients who were unable to stand up without using the armrests [28].

### 2.8. Data Analysis

After each assessment, data from the ActivPAL monitors were downloaded from a computer using PAL physical activity logging software version 7.2.38. After each assessment, transmission of data from the SENS sensor to the web server was secured by verifying that the smartphone was connected to a wireless network. The observed data (e.g., the time point for initiation and ending of each position and the number of steps taken) as well as data on gait speed and 30 sec chair stand were entered into a spreadsheet by BSP and CJO and verified by a third author (MMP). All data were transferred to and analyzed in SAS Enterprise Guide 7.1 (SAS Institute Inc., Gary, NC, USA).

Descriptive data were tested for normal distribution and are presented as mean with standard deviation (SD) or median with interquartile range (IQR) depending on the distribution of the variables. Nominal data are presented as frequencies with percentage. Data are presented based on gait speed in slow walkers (e.g., gait speed < 0.67 m/s) and fast walkers (≥0.67 m/s). A gait speed of 0.67 m/s was chosen as a cutoff since previous studies have shown the ActivPAL monitor to be accurate at measuring gait in adults walking ≥0.67 m/s [20].

The following measures of agreement were calculated as a reflection of criterion validity: (1) percentage agreement between monitors and direct observation for each patient (e.g., %of steps correctly detected = no.of steps registered by the ActivPAL or SENS∗100/no.of steps observed) for both single- and dual-sensor systems. Data are presented as mean (SD) or median (IQR) for the entire group of patients. We chose the following limits for percentage agreement:

(1) [0; 20] = very low, [20; 40] = low, [40; 60] = moderate, [60; 80] = good, and [80; 100] = high. (2) Bland-Altman plots and 95% limits of agreement (LoA) for agreement between the two monitors [29, 30] and between the monitors and direct observation for steps taken, walking time, and sedentary time (e.g., total time spent in the two lying positions) for single-sensor systems. To reflect reliability, we calculated relative (intraclass correlation coefficient (ICC_2.1_)) and absolute reliability statistics (group level: standard error of measurement (SEM and SEM%); individual level: minimal detectable change (MDC and MDC)) to illustrate the variation and measurement error between the two monitors and direct observations for steps taken, walking time, and sedentary time for single-sensor systems. Further, subgroup analyses were made for the three patient groups. ICC values of less than 0.5 were considered to reflect poor reliability, values between 0.5 and 0.75 were considered to reflect moderate reliability, values between 0.75 and 0.9 were considered to reflect good reliability, and ICCs > 0.90 were considered to reflect excellent reliability [31]. We considered a measurement error < 10% to be low, from 10% to <15% to be acceptable, and ≥15% to be high.

## 3. Results

Thirty-six patients were included in the study (Table 2). Their median age was 76 years (IQR: 72.0-82.3), 44% were female, and their median gait speed was 0.53 m/s. Twenty patients (4 older medical, 10 with acute hip fracture, and 6 after abdominal surgery) were categorized as slow walkers.

### 3.1. Percentage Agreement for Single-Sensor Systems

All data are summarized in Table 3. When used as single-sensor systems, the median (IQR) overall agreement for steps between direct observation and both monitors was good: 71.9% (39.5-80.4) for ActivPAL and 78.9% (40.3-87.1) for SENS. The overall agreement for time spent walking was good for ActivPAL (73.1% (29.3-87.6)) and high for SENS (88.9% (84.6-94.2)). The percentage agreement for steps was moderate for slow walkers and high for fast walkers for both monitors (ActivPAL: 45.9% versus 82.3%; SENS: 53.3% versus 83.6%) and for ActivPAL for walking (59.6% versus 83.9%), whereas SENS had high agreement (i.e., >80%) for all gait speeds for walking. For lying, a high median agreement was found between direct observations and both monitors (Table 4). Sitting activities were only registered by dual-sensor systems.

### 3.2. Percentage Agreement for Dual-Sensor Systems

All data are summarized in Table 4. The median (IQR) agreement for time spent sitting on a bed/chair between direct observations and ActivPAL was good (79.5% (60.9-90.2)) and high for SENS (84.6% (69.2-96.0)). For sitting in bed with the backrest elevated, the median agreement was high for ActivPAL (92.3% (46.9-100) and good for SENS (76.4% (38.9-93.2)). Results for steps, time spent walking, and standing for dual-sensor systems were equal to results for single-sensor systems for both monitors. For lying activities, SENS had high agreement for lying supine and lying with hips and knees flexed (>80%), whereas ActivPAL had good agreement (>60%) for lying supine and high agreement for lying with hips and knees flexed (>80%). As regards the sitting positions, ActivPAL was slightly better than SENS at classifying lying with elevated backrest (high versus good agreement), whereas SENS was slightly better at classifying lying supine (high versus good agreement).

### 3.3. Relative and Absolute Reliability for Steps comparisons between Observations, ActivPAL, and SENS

**Figure 1:**
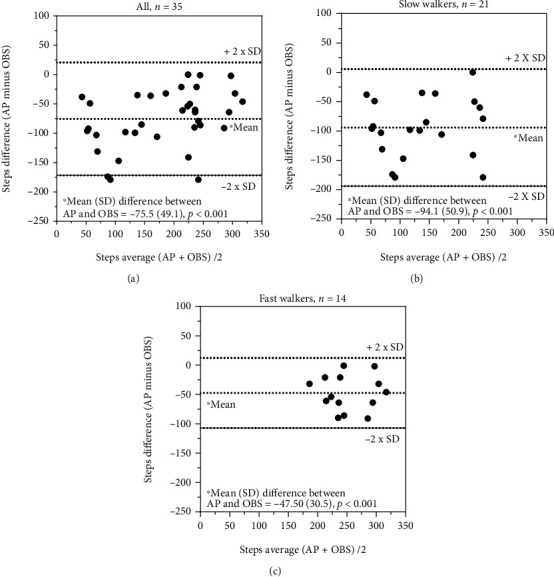
(a–c) Recording of steps: comparison between ActivPAL3™ and direct observations.

**Figure 2:**
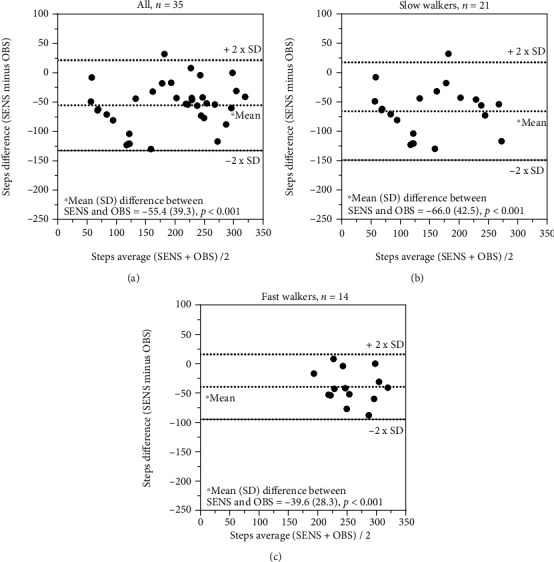
(a–c) Recording of steps: comparison between SENS motion® and direct observations.

**Figure 3:**
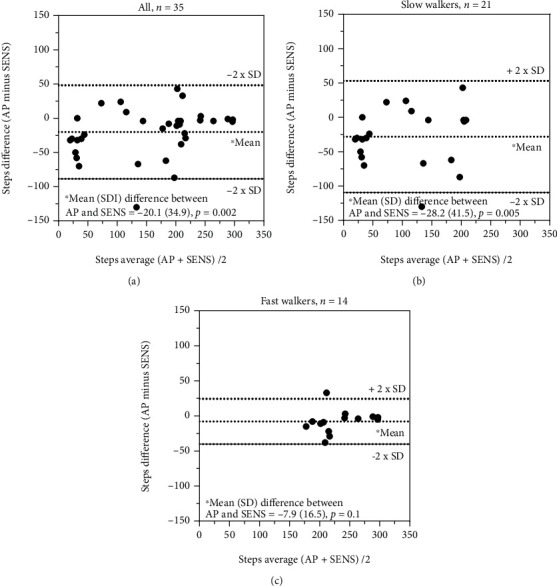
(a–c) Recording of steps: comparison between ActivPAL3™ and SENS motion®.

A systematic difference (*p* ≤ 0.005) for recording of steps (single sensors) was seen for all comparisons (Table 5, Figures 1(a)–1(c), 2(a)–2(c), and 3(a) and 3(b)), except for the comparison between ActivPAL and SENS for fast walkers (Table 5 and Figure 3(c)). The ICC_2.1_ for steps was moderate (i.e., 0.50-0.75) for all comparisons between direct observations and SENS and poor to moderate for ActivPAL, while all comparisons between ActivPAL and SENS had good reliability (i.e., 0.75-0.90) (Table 5). The corresponding measurement errors, at the group level (SEM) were high (from 18 to 39%) for overall and slow walkers and low to acceptable (5-12%) for fast walkers, while, at the individual level (MDC), this almost reached an acceptable level for fast walkers (15%) for the comparison between ActivPAL and SENS (Table 5).

### 3.4. Relative and Absolute Reliability for Walking and Sedentary Time comparisons between Observations, ActivPAL, and SENS

**Figure 4:**
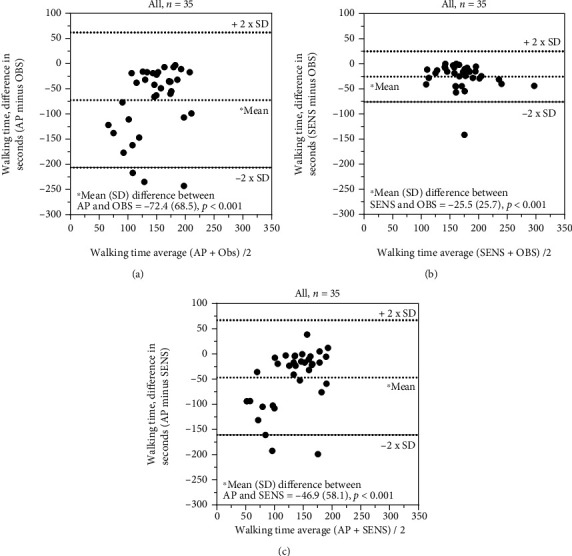
(a–c) Recording of walking time: comparison between ActivPAL3™, SENS motion®, and direct observations.

**Figure 5:**
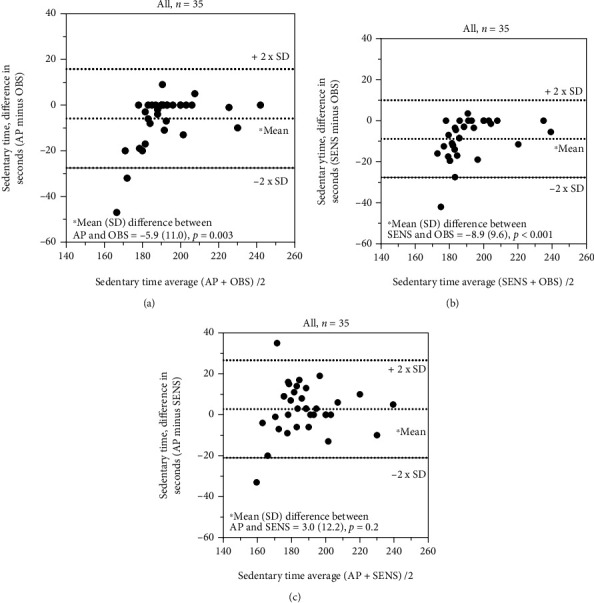
(a–c) Recording of sedentary time: comparison between ActivPAL3™, SENS motion®, and direct observations.

A systematic difference (*p* < 0.001; Table 6 and Figures 4(a)–4(c)) for recording of walking time and sedentary time (*p* ≤ 0.003; Table 6 and Figures 5(a) and 5(b)) (single sensors) was seen for all comparisons, except for the comparison between ActivPAL and SENS (Table 6 and Figure 5(c)). The ICC_2.1_ for walking and sedentary time ranged between 0.06 and 0.78, and the SEM for walking time only reached an acceptable level (13%) for the comparison between direct observations and SENS, while the MDC_95_ was >60 seconds (MDC% > 35%) for all comparisons.

Systematic (*p* ≤ 0.003), but small, differences were seen for recording of sedentary time when comparing the two monitors with direct observations (Table 6 and Figures 5(a) and 5(b)), while no systematic difference was seen between ActivPAL and SENS (Figure 5(c)). The ICC_2.1_ for sedentary time ranged between 0.72 and 0.78, with low (4%) SEM and acceptable (12%) MDC for all comparisons (Table 6).

### 3.5. Subgroup Analysis of Relative and Absolute Reliability for Steps, Walking, and Sedentary Time comparisons between Observations, ActivPAL, and SENS for the Three Patient Groups

A systematic difference (*p* ≤ 0.005) for recording of steps and walking time (single sensors) was seen for all comparisons between direct observations and both monitors for the three patient groups (older medical patients, older patients with hip fracture, and patients who underwent acute high-risk abdominal surgery). For sedentary time, a systematic difference (*p* ≤ 0.005) was seen for all comparisons between direct observation and both monitors for older medical patients and patients who underwent acute high-risk abdominal surgery; for older patients with hip fracture, a systematic difference (*p* ≤ 0.005) was seen for comparison between direct observation and SENS (Tables 7–9). The ICC_2.1_ for steps was moderate (i.e., 0.50-0.75) for all three groups (all gait speeds, fast walkers, and slow walkers) between direct observations and both ActivPAL and SENS (Tables 7–9). The corresponding measurement errors for steps at the group level (SEM) were acceptable (from 10 to 13%) for older medical patients and high (21%-55%) for older patients with hip fracture and patients who underwent acute high-risk abdominal surgery (Tables 7–9). The ICC_2.1_ for sedentary time was good (i.e., 0.75-0.9) for comparisons between direct observations and ActivPAL and for comparisons between direct observations and SENS for older patients with hip fracture and patients who underwent acute high-risk abdominal surgery (Tables 7–9). For older medical patients, the ICC_2.1_ for sedentary time was poor (<0.5) for comparisons between direct observations and both accelerometers (Tables 7–9). For walking time, the ICC_2.1_ varied from poor (<0.5) to good (0.75-0.90) for comparisons between direct observations and both ActivPAL and SENS for the three patient groups (Tables 7–9).

## 4. Discussion

The aim of this study was to examine the validity and reliability of the ActivPAL and the SENS systems compared to direct observation for time spent walking, the number of steps taken, and sedentary time. Overall, the SENS system showed stronger agreement with direct observations than ActivPAL based on median percentage agreement values. Corresponding results were seen for steps for the evaluation of measurement error at the group level, but for both monitors, the measurement error was only acceptable for fast walkers. The SENS system was better at detecting and classifying walking in slow walkers, walking slower than 0.67 m/s, with high agreement, where the ActivPAL showed moderate agreement. Still, the measurement error of both monitors in comparison with direct observations for individual persons only reached an acceptable level for the recording of sedentary time.

### 4.1. Number of Steps Taken and Walking

Gait speed influenced the accuracy of both activity monitors in the detection of number of steps taken during walking. However, the percentage agreement for time spent walking was high for SENS although not all steps were detected. A review investigating the accuracy of 15 motion sensors found all but one sensor (the Stepwatch Activity Monitor) to be inaccurate in the detection of steps at low walking speed [32]. Therefore, the ability of the SENS to perfectly assess walking time at all speeds and with an acceptable overall measurement error of 13% at the group level is promising. Previously, in patients with knee osteoarthritis, the SENS has been shown to be not reliable in the detection of walking [22]; however, a newer and improved algorithm including sporadic walking has been developed and used in this study. The results for ActivPAL are in line with previous studies [17, 21] where it also underestimated the number of steps for slow walkers. In patients walking faster than or equal to 0.67 m/s, both sensors, with a measurement error ≤ 12%, were able to more accurately classify most steps taken. For ActivPAL this agrees with previous studies where ActivPAL was able to accurately count number of steps taken and classify time spent walking in adults walking faster than or equal to 0.67 m/s [19, 20]. For the comparison of steps counted by the two monitors evaluated in the present study, the measurement error at the group level (5%) only reached an acceptable level for fast walkers. For both monitors, a predefined algorithm categorizes the recordings in predefined activities, e.g., sitting and lying down, standing, walking, and number of steps. However, both monitors have lower limits for detection of walking. A walking cadence below 0.3 Hz (ActivPAL) and 0.2 Hz (SENS) will be categorized as standing (ActivPAL) or sporadic walking (SENS), which may explain why both monitors underestimate steps and time spent walking for slow walkers. Still, assessment of physical activity and upright time (standing and walking time) in hospitalized patients (both slow and fast walking) is relevant when long periods of sedentary behavior during waking hours should be minimized.

### 4.2. Sedentary Time

Our results showed that both activity monitors, when used as single sensors, with a measurement error at the group level and individual level of only 4% and 12%, respectively, were able to perfectly classify time spent lying. This finding is in agreement with previous studies where both ActivPAL [16, 17] and SENS [22] were able to accurately classify sedentary time. However, the use of single monitors attached to the lower extremities does not allow for the distinction between different lying positions, which can be critical when, e.g., treating patients postoperatively. The supine position is associated with reduced Forced Vital Capacity (FVC) compared to upright positions and vertical positions of the torso with increased risk of pulmonary complications, such as atelectasis, pneumonia, or hypoxia [33]. Further, the supine position can impair gas exchange in the lungs [34], and oxygen saturation is better in the sitting position than in lying positions [35]. Therefore, the ability to differentiate between different lying and sitting positions can be important in hospitalized patients at risk of pulmonary complications. Consequently, we chose to include sitting in bed with elevated backrest in our protocol since we found it important to be able to classify this position as sitting in patients who spent most of their time in bed, but for whom, proper gas exchange in the lungs is important. Also, we assumed many hospitalized patients to sit in this position in their bed, and it would be preferable if the monitors did not misclassify this position with elevated backrest as lying supine. When used as dual-sensor systems, both monitors were able to accurately classify time spent sitting on the bed and time spent sitting in bed with the backrest elevated; however, when used as dual sensors, both activity monitors less accurately classified lying supine but accurately classified lying supine with knees and hips flexed. Therefore, depending on the purpose of evaluating patient activity two monitors, as opposed to one, can be relevant as an activity indicator for health care professionals. Also, the use of activity monitors as feedback has been shown to have a positive effect on patient activity levels [13, 36].

## 5. Strengths and Limitations

### 5.1. Strengths

The strength of this study is that the accuracy of the activity monitors was a5ssessed with standardized activities and direct observations of the activities as reference criteria. Lindemann et al. [37] recommend following a standardized protocol to detect activities and using either video observations or direct observations as reference criteria. In addition, the two set of sensors were placed on the chest and thigh in all patients according to the assessment protocol to ensure that different sensor placements would not affect the outcome. Also, we examined the activity monitors as both single- and dual-sensor systems to be able to evaluate the accuracy of both walking, standing, sitting, and lying positions and thereby open up for the use of sensors in patients being bedbound, for whom positioning is critical. Lastly, we examined the activity monitors in three different in-patient populations to increase the generalizability of the findings.

### 5.2. Limitations

The study has some limitations worth considering. Firstly, the sensors were evaluated within a limited time and following a strict protocol. An evaluation of the sensors over, e.g., an entire day and in more free-living situations, could have been useful. However, this was a pragmatic solution since we chose direct observation as reference criteria. Secondly, we only evaluated sitting in bed with the backrest elevated 60^○^. It could have been relevant to evaluate different inclinations of the bedrest, but the cutoff of 60^○^ was chosen since this was the cutoff incorporated in the dual-sensor algorithm of the SENS-monitors. Thirdly, a different placement of the leg sensors may have enhanced the validity of the measurements since placement of sensors can affect output [38]. For example, we could have placed the sensors on the ankle to enhance step sensitivity. A study by Bezuidenhout et al. [39] on ActiGraph monitors has shown ankle placement to be more sensitive in measuring steps than hip worn placement monitors. However, we chose to place the monitors on the thigh as this is the placement suggested by the developers of both ActivPAL and SENS.

## 6. Conclusion

Both the ActivPAL3™ and the SENS motion® system showed good to high percentage agreement with direct observation for standing, sitting, lying, walking (all gait speeds), and no. of steps (all gait speeds). Relative reliability for steps was poor to moderate for comparisons between direct observations and ActivPAL3™ and moderate for all comparisons between direct observations and SENS motion®. The SENS motion® system showed slightly less overall measurement error with the observed number of steps than ActivPAL3™, but both monitors reached an acceptable level of reliability for fast walkers only. In patients walking ≥0.67 m/s, both sensors reached a good to high agreement in classifying both number of steps and walking. Only SENS motion® reached a good agreement for walking time in patients walking slower than 0.67 m/s.

## Figures and Tables

**Table 1:** Standardized activities.

Activity	Duration
Lying supine, backrest flat	1 min
Lying supine, hips and knees flexed	1 min
Sitting in bed, legs straight and backrest 60^○^	1 min
Sitting on a bed	1 min
Sitting on a standard chair (height 45 cm)	1 min
Standing	1 min
Walking	3 min
No. of steps	Assessed during walking

**Table 2:** Patient characteristics.

Variables	Overall	Older medical patients	Older patients with acute hip fracture	Acute high-risk abdominal surgery patients
Age, median (IQR) (range) (*n*)	76.0 (72.0-82.3) (28-92) (*n* = 36)	74.5 (72.5-77.0) (67-91) (*n* = 12)	81.5 (75.8-85.5) (72-92) (*n* = 12)	70.0 (57.0-78.5) (28-92) (*n* = 12)
Sex, female; *n* (%)	16 (44.0)	3 (25.0)	8 (67.0)	5 (42.0)
Gait speed (m/s), median (IQR) (*n*)	0.53 (0.32-0.86) (*n* = 35^∗^)	0.77 (0.52-1.04) (*n* = 12)	0.27 (0.22-0.39) (*n* = 11^∗^)	0.67 (0.50-0.95) (*n* = 12)
Slow walkers, *n* (%)	20 (57.0)	4 (20.0)	10 (50.0)	6 (30.0)
Fast walkers, *n* (%)	15 (43.0)	8 (53.0)	1 (7.0)	6 (40.0)
30 s CST, median (IQR)	10 (7-10) (*n* = 14)	10 (10-11) (*n* = 6)		9 (7-10) (*n* = 8)
30 s CST mod, median (IQR)	4 (3-7) (*n* = 22)	8 (4-11) (*n* = 6)	3 (2-4) (*n* = 12)	4 (3-6) (*n* = 4)

^∗^One patient with acute hip fracture could not finish the 4-meter gait speed test due to pain; slow walkers: gait speed < 0.67 m/s; fast walkers: gait speed ≥ 0.67 m/s; 30 s CST: 30-second chair-stand test; 30 s CST mod: modified 30-second chair-stand test (use of armrests to perform the test).

**Table 3:** Agreement between steps and walking measured by direct observations and by ActivPAL3™ and SENS motion® used as both single- and dual-sensor systems.

	Steps			Walking time		
*Assessment method*
	All gait speeds *N* = 35	Slow walkers^∗∗^ *N* = 20	Fast walkers^∗∗∗^ *N* = 14	All gait speeds *N* = 35	Slow walkers^∗∗^ *N* = 20	Fast walkers^∗∗∗^ *N* = 14^#^
Direct observation	7738^∗^	3811^∗^	3865^∗^	6397^#^	3758^#^	2446^#^
ActivPAL, single sensor	5096^∗^	1872^∗^	3200^∗^	3863^#^	1778^#^	2039^#^
SENS, single sensor	5800^∗^	2311^∗^	3311^∗^	5506^#^	3175^#^	2183^#^
ActivPAL, dual sensor	5096^∗^	1872^∗^	3200^∗^	3795^#^	1725^#^	2040^#^
SENS, dual sensor	5800^∗^	2435^∗^	3311^∗^	5506^#^	3175^#^	2182^#^
*Percentage agreement between direct observation and ActivPAL/SENS*						
ActivPAL (single sensor), median % (IQR)	71.9 (39.5-80.4)	45.9 (10.7-74.7)	82.3 (75.1-90.6)	73.1 (29.3-87.6)	59.6 (9.4-82.4)	83.9 (73.1-92.1)
SENS (single sensor), median % (IQR)	78.9 (40.3-87.1)	53.33 (38.8-81.4)	83.6 (78.4-91.4)	88.9 (84.6-94.2)	89.4 (81.3-92.6)	89.4 (86.3-95.7)
ActivPAL (dual sensor), median % (IQR)	71.9 (39.5-80.4)	45.9 (10.7-74.7)	82.3 (75.1-90.6)	73.1 (29.3-87.6)	59.2 (9.3-82.4)	83.0 (73.9-95.1)
SENS (dual sensor), median % (IQR)	78.9 (40.3-87.1)	53.33 (38.8-81.4)	83.6 (78.4-91.4)	88.9 (84.6-94.2)	89.4 (81.3-92.6)	89.4 (86.3-95.7)

^∗^Number of steps; ^#^walking time in sec; ^∗∗^slow walkers: <0.67 m/s; ^∗∗∗^fast walkers: ≥0.67 m/s. ActivPAL: ActivPAL3™ monitor; SENS: SENS motion®.

**Table 4:** Agreement between standing, sitting, and lying measured by direct observations and by ActivPAL3™ and SENS motion® used as both single- and dual-sensor systems.

	Standing	Lying		Sitting	
*Assessment method*	Standing *N* = 35	Supine *N* = 35	Flexed hips & knees *N* = 35	On bed/chair *N* = 35	In bed, backrest elevated 60° *N* = 35
Direct observation (sec)	2215	2281	2249	4774	2281
ActivPAL, single sensor (sec)	1507	2137	2184		
SENS, single sensor (sec)	1396	2339	2161		
ActivPAL, dual sensor (sec)	1505	1665	2118	3401	1580
SENS, dual sensor (sec)	1396	1808	2144	3740	1478
*Percentage agreement between direct observation and ActivPAL/SENS*					
ActivPAL (single sensor), median % (IQR)	73.5 (47.8-90.6)	98.4 (89.6-100)	100 (95.2-100)		
SENS (single sensor), median % (IQR)	67.7 (43.4-85.9)	99.2 (94.5-100)	99.2 (94.5-100)		
ActivPAL (dual sensor), median % (IQR)	71.4 (47.6-95.2)	78.1 (50.0-98.4)	100 (93.8-100)	79.5 (60.9-90.2)	92.3 (46.9-100)
SENS (dual sensor), median % (IQR)	67.7 (43.4-85.9)	93.1 (55.9-99.2)	99.2 (94.5-100)	84.6 (69.2-96.0)	76.4 (38.9-93.2)

ActivPAL: ActivPAL3™ monitor; SENS: SENS motion®.

**Table 5:** Relative and absolute reliability of steps, assessed with ActivPAL3™, SENS motion®, and direct observations.

Comparison	Mean (SD) of both assessments (steps)	Mean (SD) difference (steps)	*p* value for difference	ICC_2.1_ (95% CI)	SEM (SEM%)	MDC_95_ (MDC%)
ActivPAL vs. observed						
All, *N* = 35	183.3 (82.2)	-75.5 (49.1)	<0.001	0.609 (-0.086-0.865)	51.4 (28)	142.5 (78)
Slow walkers, *n* = 21	137.4 (71.2)	-94.1 (50.9)	<0.001	0.439 (-0.095-0.791)	53.3 (39)	147.7 (107)
Fast walkers, *n* = 14	252.3 (40.0)	-47.5 (30.5)	<0.001	0.468 (-0.110-0.820)	29.2 (12)	80.9 (32)
SENS vs. observed						
All, *N* = 35	193.4 (78.6)	-55.3 (39.3)	<0.001	0.717 (-0.043-0.909)	41.8 (22)	115.9 (60)
Slow walkers, *n* = 21	151.5 (70.1)	-65.9 (42.5)	<0.001	0.596 (-0.093-0.870)	44.6 (29)	123.6 (82)
Fast walkers, *n* = 14	256.3 (38.3)	-39.5 (28.4)	<0.001	0.523 (-0.106-0.845)	26.5 (10)	73.5 (29)
ActivPAL vs. SENS						
All, *N* = 35	155.7 (88.5)	-20.1 (34.8)	0.002	0.905 (0.758-0.957)	27.3 (18)	75.7 (49)
Slow walkers, *n* = 21	104.4 (73.4)	-28.3 (41.4)	0.005	0.803 (0.451-0.925)	32.6 (31)	90.4 (89)
Fast walkers, *n* = 14	232.6 (40.0)	-8.0 (16.5)	0.1	0.906 (0.723-0.969)	12.3 (5)	34.1 (15)

ICC: intraclass correlation coefficient; SEM: standard error of measurement (SD of mean [both assessments] × √1 − ICC); MDC: minimal detectable change (SEM × √2 × 1.96, SEM% = 100 × SEM/mean). SEM and MDC indicate measurement error (in sec) at group and individual levels, respectively. Slow walkers: <0.67 m/s; fast walkers: ≥0.67 m/s. ActivPAL: ActivPAL3™ monitor; SENS: SENS motion®.

**Table 6:** Relative and absolute reliability of walking and sedentary time in seconds, assessed with ActivPAL3™, SENS motion®, and direct observations.

Comparison	Mean (SD) of both assessments (sec)	Mean (SD) difference (sec)	*p* value for difference	ICC_2.1_ (95% CI)	SEM (SEM%)	MDC_95_ (MDC%)
Walking time						
ActivPAL vs. observed	146.6 (38.6)	-72.4 (68.5)	<0.001	0.059 (-0.104-0.272)	37.4 (26)	80.9 (55)
SENS vs. observed	170.0 (37.6)	-25.5 (25.7)	<0.001	0.660 (0.079-0.863)	21.9 (13)	60.7 (36)
ActivPAL vs. SENS	133.8 (40.3)	-46.9 (58.1)	<0.001	0.222 (-0.069-0.496)	35.5 (27)	98.4 (74)
Sedentary time						
ActivPAL vs. observed	192.3 (15.8)	-5.9 (11.0)	0.003	0.740 (0.479-0.870)	8.1 (4)	22.5 (12)
SENS vs. observed	190.8 (15.4)	-8.9 (9.6)	<0.001	0.718 (0.179-0.888)	8.2 (4)	22.7 (12)
ActivPAL vs. SENS	187.9 (17.4)	3.0 (12.2)	0.159	0.776 (0.601-0.880)	8.2 (4)	22.7 (12)

ICC: intraclass correlation coefficient; SEM: standard error of measurement (SD of mean [both assessments] × √1 − ICC); MDC: minimal detectable change (SEM × √2 × 1.96, SEM% = 100 × SEM/mean). SEM and MDC indicate measurement error (in sec) at group and individual levels, respectively. ActivPAL: ActivPAL3™ monitor; SENS: SENS motion®.

**Table 7:** Relative and absolute reliability of steps, walking, and sedentary time assessed with ActivPAL3™, SENS motion®, and direct observations in older medical patients (*N* = 12).

Comparison	Mean (SD) of both assessments (sec)	Mean (SD) difference (sec)	*p* value for difference	ICC_2.1_ (95% CI)	SEM (SEM%)	MDC_95_ (MDC%)
Steps						
ActivPAL vs. observed	229.1 (45.8)	-43.6 (29.6)	<0.001	0.582 (-0.103-0.882)	29.6 (13)	82.0 (36)
SENS vs. observed	235.2 (41.1)	-31.5 (23.0)	0.001	0.678 (-0.076-0.916)	23.3 (10)	64.6 (27)
ActivPAL vs. SENS	213.4 (47.9)	-12.1 (24.8)	0.1	0.858 (0.579-0.957)	18.1 (8)	59.2 (24)
Walking time						
ActivPAL vs. observed	160.0 (26.4)	-25.8 (16.3)	<0.001	0.582 (-0.102-0.884)	17.1 (11)	47.4 (30)
SENS vs. observed	165.8 (25.1)	-14.2 (9.0)	<0.001	0.816 (-0.038-0.960)	10.8 (7)	29.9 (18)
ActivPAL vs. SENS	152.9 (25.5)	-11.5 (12.7)	0.009	0.813 (0.243-0.950)	11.0 (7)	30.5 (20)
Sedentary time						
ActivPAL vs. observed	184.8 (9.8)	-9.3 (10.7)	0.012	0.416 (-0.092-0.778)	7.5 (4)	20.8 (11)
SENS vs. observed	183.4 (9.2)	-12.0 (11.9)	0.005	0.264 (-0.141-0.671)	7.9 (4)	21.9 (12)
ActivPAL vs. SENS	178.8 (12.3)	2.8 (13.6)	0.5	0.544 (-0.015-0.844)	8.3 (5)	23.0 (13)

ICC: intraclass correlation coefficient; SEM: standard error of measurement (SD of mean [both assessments] × √1 − ICC); MDC: minimal detectable change (SEM × √2 × 1.96, SEM% = 100 × SEM/mean). SEM and MDC indicate measurement error (in sec) at group and individual levels, respectively. ActivPAL: ActivPAL3™ monitor; SENS: SENS motion®.

**Table 8:** Relative and absolute reliability of steps, walking, and sedentary time assessed with ActivPAL3™, SENS motion®, and direct observations in older patients with hip fracture (*N* = 12).

Comparison	Mean (SD) of both assessments (sec)	Mean (SD) difference (sec)	*p* value for difference	ICC_2.1_ (95% CI)	SEM (SEM%)	MDC_95_ (MDC%)
Steps						
ActivPAL vs. observed	124.6 (83.7)	-95.8 (48.8)	<0.001	0.530 (-0.089-0.868)	57.4 (46)	159.1 (128)
SENS vs. observed	139.3 (83.4)	-66.3 (48.4)	0.001	0.660 (-0.081-0.910)	48.2 (35)	133.6 (96)
ActivPAL vs. SENS	91.4 (82.5)	-29.5 (44.4)	0.042	0.825 (0.431-9.949)	14.4 (16)	39.9 (44)
Walking time						
ActivPAL vs. observed	140.3 (47.5)	-126.5 (78.5)	<0.001	0.062 (-0.097-0.375)	46.0 (33)	127.5 (91)
SENS vs. observed	184.5 (54.8)	-38.1 (36.8)	0.004	0.664 (-0.003-0.903)	31.8 (17)	88.1 (48)
ActivPAL vs. SENS	121.3 (51.5)	-88.4 (63.2)	0.001	0.223 (-0.121-0.632)	45.4 (37)	125.8 (103)
Sedentary time						
ActivPAL vs. observed	196.5 (12.1)	1.1 (2.9)	0.2	0.970 (0.903-0.991)	2.1 (1)	5.8 (3)
SENS vs. observed	192.9 (11.3)	-6.0 (6.6)	0.010	0.756 0.157-0.932()	5.6 (3)	15.5 (8)
ActivPAL vs. SENS	193.5 (12.1)	7.0 (6.7)	0.004	0.716 (0.030-0.933)	6.4 (3)	17.7 (9)

ICC: intraclass correlation coefficient; SEM: standard error of measurement (SD of mean [both assessments] × √1 − ICC); MDC: minimal detectable change (SEM × √2 × 1.96, SEM% = 100 × SEM/mean). SEM and MDC indicate measurement error (in sec) at group and individual levels, respectively. ActivPAL: ActivPAL3™ monitor; SENS: SENS motion®.

**Table 9:** Relative and absolute reliability of steps, walking, and sedentary time assessed with ActivPAL3™, SENS motion®, and direct observations in patients following acute high-risk abdominal surgery (*N* = 11).

Comparison	Mean (SD) of both assessments (sec)	Mean (SD) difference (sec)	*p* value for difference	ICC_2.1_ (95% CI)	SEM (SEM%)	MDC_95_ (MDC%)
Steps						
ActivPAL vs. observed	197.5 (80.2)	-88.1 (52.3)	<0.001	0.529 (-0.105-0.868)	55.0 (28)	152.5 (77)
SENS vs. observed	206.9 (75.3)	-69.4 (32.3)	<0.001	0.653 (-0.075-0.920)	44.4 (21)	123.0 (59)
ActivPAL vs. SENS	162.8 (87.5)	-18.7 (33.2)	0.09	0.916 (0.699-0.977)	25.4 (16)	70.4 (43)
Walking time						
ActivPAL vs. observed	138.7 (38.1)	-64.2 (51.7)	0.002	0.193 (-0.135-0.612)	34.2 (25)	94.8 (68)
SENS vs. observed	158.9 (20.4)	-23.9 (18.0)	0.001	0.435 (-0.121-0.812)	15.3 (10)	42.4 (27)
ActivPAL vs. SENS	126.7 (34.9)	-40.4 (57.6)	0.042	0.143 (-0.264-0.610)	32.3 (25)	89.5 (71)
Sedentary time						
ActivPAL vs. observed	195.9 (21.8)	-9.8 (13.8)	0.040	0.760 (0.263-0.932)	10.7 (5)	29.7 (15)
SENS vs. observed	196.5 (21.6)	-8.6 (9.4)	0.012	0.853 (0.309-0.964)	8.3 (4)	22.2 (11)
ActivPAL vs. SENS	191.6 (23.9)	-1.2 (14.7)	0.8	0.840 (0.506-0.954)	9.6 (5)	26.6 (14)

ICC: intraclass correlation coefficient; SEM: standard error of measurement (SD of mean [both assessments] × √1 − ICC); MDC: minimal detectable change (SEM × √2 × 1.96, SEM% = 100 × SEM/mean). SEM and MDC indicate measurement error (in sec) at group and individual levels, respectively. ActivPAL: ActivPAL3™ monitor; SENS: SENS motion®.

## Data Availability

The datasets generated and/or analyzed during the current study are not publicly available due to regulations set out by the Danish Data Protection Agency.

## Supplementary Materials

Supplementary Materials Assessment protocol—standardized activities and attachment of sensors.
