# Supplementary material for: Validation of Two Activity Monitors in Slow and Fast Walking Hospitalized Patients
Source: Rehabil Res Pract. 2022 May 16;2022:9230081. doi: 10.1155/2022/9230081 (PMC9126721; doi:10.1155/2022/9230081)
Supplement: Supplementary Materials — Assessment protocol—standardized activities and attachment of sensors. [file 9230081.f1.docx]

**Assessment protocol - Standardized activities and attachment of sensors**

1. **Attachment of ActivPAL and SENS**

Prior to attachment of sensors, the patient will be informed about all body positions and activities and the order of these. Hereafter, the patient will be asked to lie down in a supine position with the head of the bed elevated to 60 degrees and the sensors will be attached.

**SENS-motion lower extremity**

Right thigh: On the line between the lateral epicondyle of femur and trochanter major, mark a point 10 cm proximal to the lateral epicondyle. Place a sensor with the teardrop shaped band aid pointing downwards and parallel to the iliotibial band. Mark the band aid with the letters “LE”. In patients with hip fractures, place the sensor on the contralateral leg.

**SENS-motion thorax**

Palpate the sternal end of the right clavicle and mark a point 2 cm distally from here. Place a sensor on the marked point with the teardrop shape pointing downwards. Mark the band aid with the letter: “T”.

For patients with left side hip-fractures, the sternal sensor is placed distally to the left clavicle.

**ActivPAL lower extremity**

Right thigh: On the line between the base of the patella and the spina illiaca anterior superior (SIAS), mark a point 1/3 of the of the distance between SIAS and patella (downwards from SIAS). Place an activPAL sensor vertically on this point so that the person on the ActivPAL sensor is standing upright. Mark the ActivPAL sensor with the letters: “LE”. For patients with hip fractures, place the sensor on the contralateral leg.

**ActivPAL thorax**

Palpate the sternal end of the left clavicle and mark a point 2 cm distally from here. Place a sensor laterally on the marked point with the person on the sensor standing upright. Mark the band aid with the letter: “T”.

For patients with left side hip-fractures, the sternal sensor is placed distally to the right clavicle.

## Activation of SENS motion and *Activ*PAL

Note the time of activation, attachment, detachment and deactivation of the sensors below.

| SENS LE (lower extremity) | | |
| --- | --- | --- |
| Sensor activated | Date ____________ | Time _______________ (Hour: minute: sec.) |
| Sensor attached | Date ____________ | Time _______________ (Hour: minute: sec.) |
| Sensor detached | Date ____________ | Time _______________ (Hour: minute: sec.) |
| Sensor deactivated | Date ____________ | Time _______________ (Hour: minute: sec.) |
| SENS T Thorax | | |
| Sensor activated | Date ____________ | Time _______________ (Hour: minute: sec.) |
| Sensor attached | Date ____________ | Time _______________ (Hour: minute: sec.) |
| Sensor detached | Date ____________ | Time _______________ (Hour: minute: sec.) |
| Sensor deactivated | Date ____________ | Time _______________ (Hour: minute: sec.) |

| ActivPal LE (lower extremity) | | |
| --- | --- | --- |
| Sensor activated | Date ____________ | Time _______________ (Hour: minute: sec.) |
| Sensor attached | Date ____________ | Time _______________ (Hour: minute: sec.) |
| Sensor detached | Date ____________ | Time _______________ (Hour: minute: sec.) |
| Sensor deactivated | Date ____________ | Time _______________ (Hour: minute: sec.) |
| **ActivPal T Thorax** | | |
| Sensor activated | Date ____________ | Time _______________ (Hour: minute: sec.) |
| Sensor attached | Date ____________ | Time _______________ (Hour: minute: sec.) |
| Sensor detached | Date ____________ | Time _______________ (Hour: minute: sec.) |
| Sensor deactivated | Date ____________ | Time _______________ (Hour: minute: sec.) |

1. **Observations and test times for positions**

Note the activation time of the sensors when they are activated via the laptop. Ask the patient to perform all positions in the randomized order.

**Tester 1:**

Notes the start and end time of each position in minutes and seconds. The start time is defined as the first time the patient assumes the new position.

**Tester 2**:

Informs the patient about when to change position and prepares the patient for the upcoming position well in advance.

| Overall time of start of observations | |
| --- | --- |
| Joint time of start for observations (Tester 1): | Start time_______________ (Hour: minute)  Stopwatch time___________ (Hour: minute: sec.) |

| 1. | Supine position | | |
| --- | --- | --- | --- |
| 1 min. | Supine position in the bed  Head end elevated to 60 degrees | Start time:______________ | End time:____________ |
| 1 min. | Supine position in the bed | Start time:______________ | End time:____________ |
| 1 min. | Supine position in the bed  with hips and knees bent and feet in the ground/mat (hips flexed to 80 degrees) | Start time:______________ | End time:____________ |
| 1 min. | Transfer to the next position |  |  |

| 2. | Sitting position on the bed | | |
| --- | --- | --- | --- |
| 1 min. | Sitting on the bedside | Start time:______________ | End time:____________ |
| 1 min. | Transfer to the next position |  |  |

| 3. | Standing position | | |
| --- | --- | --- | --- |
| 1 min. | Standing (by the bedside) | Start time:______________ | End time:____________ |
| 1 min. | Transfer to the next position |  |  |

| 4. | Walking | | |
| --- | --- | --- | --- |
|  | **Instruction**  *In a moment, you must start walking. You must walk for 3 minutes at your own pace and only stop when I tell you to or when you feel you cannot walk any longer* (Note the use of walking aids). | | |
| 3 min. | Walking | Start time:______________  Numbers of steps:________ | End time:____________ |
|  | Walking aids: | No walking aids  Walking stick  Elbow sticks  Rollator walker  Other _______________ | ****  ****  ****  ****  **** |

| 5. | Sitting position on a chair | | |
| --- | --- | --- | --- |
| 1 min. | Sitting on a chair | Start time:______________ | End time:____________ |
| 1 min. | Transfer to the next position |  |  |

| Overall end time for the observations | |
| --- | --- |
| Overall end time for the observations (Tester 1): | End time at______________(Hour: minut)  Stopwatch time __________(Hour: minut: sec.) |

1. **Walking pace**

| **4 meter walking test** | | | | | |
| --- | --- | --- | --- | --- | --- |
| **Instruction**  *Now, I will observe you walking. If you use a stick or another walking aid and you feel the need to use a walking aid for this walking test, you are welcome to do so. This is the distance you have to walk.* (Shows the walking distance, from point a to past point b).  *You have to walk the distance at your normal pace, just like when you walk down the street.*  *You must walk the entire distance past point b. I’ll walk with you when you walk.* (Walking distance is demonstrated to the patient - the tester walks past point b and then stops).  (The patient is instructed to be ready at point a). *When you have to start walking, I will say "ready start"*. (When the patient is ready, the instruction “ready start” is given, and the stopwatch is started when the patient starts walking and one foot crosses point a. Walk next to or behind the patient. Stop the stopwatch when the patient passes point b with one foot). | | | | | |
| Walking aids | No walking aids  Walking stick  Elbow sticks  Rollator walker  Other________________ | | ****  ****  ****  ****  **** | |  |
| Footwear | No footwear  Flat shoes  High shoes | | ****  ****  **** |  | |
| Results | Test 1  Test 2 | | ____,___sec  ____,___sec | The stopwatch is started when the patient starts walking and one foot crosses point a. Stop the stopwatch when the patient passes point b with one foot. | |
| Fastest result of tests 1 and 2 | |  | _____,___sec |  | |

**The test was not carried out because:**

 Does not wish to participate.

 Unable to attend due to physical inconveniences, for example due to pain.

 The patient cannot cooperate.

 Unable to attend due to acute worsening of current condition.

1. **Sit-to-stand**

| **Sit-to-stand (a chair with a 45 cm seat height)** | |
| --- | --- |
| **Instruction**  *Now we will find out how strong your legs are by testing how many times you can get up from a chair and sit down again in 30 sec. I will show you how it is done.* (Show how the test is carried out, start slowly to demonstrate the technique). *Sit down on the middle of the chair (with your back straight) and your feet parallel and next to each other. Cross your wrists and hold them against your chest. It is important that you get up completely and sit down completely, otherwise it does not count. When I say “ready-set-GO”, get up and sit down as many times as possible for 30 sec.* (Show the test at a fast pace so that the patient is aware of how fast to do it).  Modified sit-to-stand: Applied for patients with hip fracture or if the patient cannot carry out the exercise ("get up-sit-down") without the use of an armrests. Show the test (sit-to-stand. Start at a slow pace to demonstrate the technique. *You need to lean on the armrests when you get up. It is important that you stand up completely (standing straight) every time, otherwise it does not count.* *When I say “ready-set-GO”, get up and sit down as many times as possible for 30 sec.* | |
| Is the participant able to get up once without the use of armrests? Seat height | Yes  No   _______________ cm |
| Sit-to-stand | ____________ number of repetitions in 30 sec |
| Modified sit-to-stand | ___________ number of repetitions in 30 sec |

**The test was not carried out because:**

 Does not wish to participate.

 Unable to attend due to physical inconveniences, for example due to pains.

 The patient cannot cooperate.

 Unable to attend due to acute worsening of current condition.

## Ending

|  | |
| --- | --- |
| Total end time for observations and tests (Tester 1): | Time _____________________ (Hour: minute: sec.) |
